# ML for fast assimilation of wall-pressure measurements from hypersonic flow over a cone

**DOI:** 10.1038/s41598-024-63053-4

**Published:** 2024-06-04

**Authors:** Pierluigi Morra, Charles Meneveau, Tamer A. Zaki

**Affiliations:** Department of Mechanical Engineering, Johns Hopkins University, 3400 N. Charles Street, Baltimore, MD 21218 USA

**Keywords:** Deep operator networks, Hypersonics, Data assimilation, Engineering, Aerospace engineering, Mechanical engineering

## Abstract

Data assimilation (DA) integrates experimental measurements into computational models to enable high-fidelity predictions of dynamical systems. However, the cost associated with solving this inverse problem, from measurements to the state, can be prohibitive for complex systems such as transitional hypersonic flows. We introduce an accurate and efficient deep-learning approach that alleviates this computational burden, and that enables approximately three orders of magnitude computational acceleration relative to variational techniques. Our method pivots on the deployment of a deep operator network (DeepONet) as an accurate, parsimonious and efficient meta-model of the compressible Navier–Stokes equations. The approach involves two main steps, each addressing specific challenges. Firstly, we reduce the computational load by minimizing the number of costly direct numerical simulations to construct a comprehensive dataset for effective supervised learning. This is achieved by optimally sampling the space of possible solutions. Secondly, we expedite the computation of high-dimensional assimilated solutions by deploying the DeepONet. This entails efficiently navigating the DeepONet’s approximation of the cost landscape using a gradient-free technique. We demonstrate the successful application of this method for data assimilation of wind-tunnel measurements of a Mach 6, transitional, boundary-layer flow over a 7-degree half-angle cone.

## Introduction

Design of hypersonic vehicles is challenging in part due to the requirement that the vehicle sustains the thermo-mechanical loads. Prediction of design performance is especially difficult when the flow around the vehicle is transitional, i.e. transitions from a laminar to a turbulent state. Transition takes place in the boundary layer, which is a very thin region in the immediate vicinity of the vehicle where flow quantities vary rapidly from their surface to edge values. When the boundary layer becomes turbulent, surface heat-flux and skin friction dramatically increase. Failing to account for these factors in the design of a vehicle with a desired mission duration of minutes can have catastrophic results, as evidenced by the X-15A crash in 1967^1^. Transitional high-speed flows are difficult to study experimentally and computationally, due to the very short time- and length-scales of the instability waves and, most importantly, due to the sensitivity of flow instabilities to uncertainties^2^. To tackle this engineering and scientific challenge, experiments and simulations have traditionally been adopted as complementary tools, each having strengths that mitigate the shortcomings of the other. Significant progress can be made by the use of data assimilation (DA), which solves a challenging, nonlinear, inverse problem whereby the computational predictions are optimized to reproduce available experimental measurements. DA rids the simulations of *ad hoc* assumptions and increases its fidelity, and augments the available measurements with trustworthy computational predictions. In this work, after a review of existing methods, we put forward the idea of using deep operator networks (DeepONet) to accelerate data assimilation in transitional hypersonic flows, and we address the key computational challenges associated with adopting these networks.

Flight tests are the most valuable source of hypersonic, transitional flow data^3–6^. However, these tests are expensive, the flow environment is uncertain, and the measurements are limited. As a result, flight tests are not easily repeated nor are they necessarily reproducible. Laboratory experiments address some of these difficulties by providing more controlled environments and accommodating measurements techniques that are not possible in flight^7–10^. They are also reproducible and can generate more data, although the measurements remain limited in spatio-temporal resolution and their interpretation is difficult because of tunnel uncertainties^11^. Upstream measurements in the early stages of linear flow instabilities are difficult to perform due to the small amplitudes of the disturbance waves, and downstream measurements in the nonlinear regime are more challenging to interpret. In addition, some flow quantities are impossible to measure directly.

Unlike experiments, computational models enable non-intrusive access to all flow quantities of interest^12–15^. The shortcoming of computations, even direct numerical simulations (DNS) that resolve all the flow scales, is that they invoke modeling assumptions, most importantly idealized inflow/boundary conditions that determine the downstream flow, including laminar-to-turbulence transition^16^. Such idealizations are a main source of uncertainty since changing the oncoming waves can quantitatively, and also qualitatively, change the transition process^16–20^.

The field of data assimilation (DA) fuses experiments and simulations. Available measurements are adopted as targets that computations aim to reproduce quantitatively. By adjusting uncertain parameters until their optimal values are discovered, simulations become significantly more realistic. Such computations then provide non-intrusive access to all flow quantities of interest.

In fluid dynamics, DA has been applied to a wide range of flow regimes using various strategies, including filtering and nudging^21–25^, adjoint-variational techniques^26–30^, ensemble-variational methods^31–34^ and neural networks^35–42^. In the context of high-speed, transitional boundary layers, the most relevant work for our purposes is by Buchta et al.^34^. They employed an ensemble-variational (EnVar) technique to deduce the upstream perturbations from downstream wall-pressure measurements on a cone. Their recent investigation was the first-of-its-kind to accurately interpret sensor data from within the nonlinear, transitional flow regime. A critical aspect to bear in mind is that for each new set of measurements, the EnVar process must be repeated. Pre-trained neural networks, capable of swift evaluation hold the promise of expediting the solution of these inverse problems.

**Figure 1:**
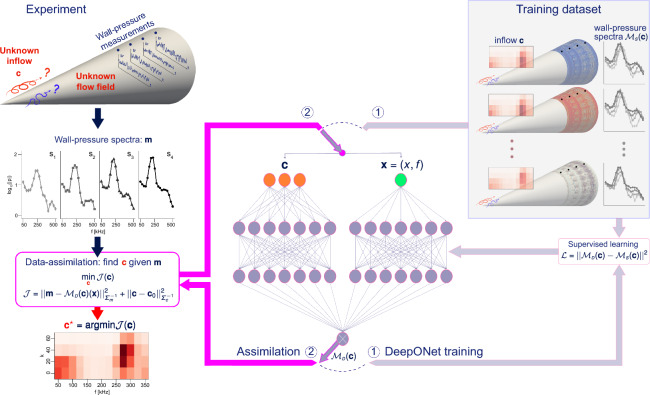
Schematic of the proposed method, FastDA (Fast assimilation through DeepONet acceleration), consisting of two loops. DeepONet training loop (1): train the neural network only once, with available DNS data. Fast assimilation loop (2): search the inflow $\varvec{c}$ which results in the experimental measurements $\varvec{m}$ with a DeepONet as meta-model of the compressible Navier–Stokes equations.

In this work, we introduce a strategy that exploits deep learning to expedite the interpretation of experimental observations. The measurements are wall-pressure spectra acquired from PCB sensors on a 7-degree cone, at free-stream Mach number $M=6.14$, as in Buchta et al.^34^. We estimate the upstream instability waves, quantitatively reproduce the wall-pressure spectra, and discover the full spatio-temporal flow field that led to the measured data. We compare our results with the recent and only known solution, by Buchta et al.^34^, and show that within the time needed for EnVar to solve one data assimilation problem our approach computes about 2000 solutions, with commensurate accuracy.

The flow configuration of interest and the essence of our proposed strategy are presented schematically in Fig. 1. We are provided with experimental measurements from Mach $M=6.14$ flow over a $7^{\circ }$ half-angle cone. The measurements are the signals from four PCB pressure sensors ($s_1, s_2, s_3, s_4$), flush-mounted on the surface of a cone. As depicted in the schematic, we are not provided any information regarding the spatio-temporal details of the flow, and rely solely on the measurements from these four probes. Using Welch’s method, we analyze these experimental signals and evaluate their spectral content. The spectral information is then adopted in a data assimilation process to discover the unknown oncoming disturbances. Once discovered, using the upstream conditions as input enables us to simulate and to accurately predict the entire flow which is consistent with the measurements, and thus to have access to the full spatio-temporal fields. Further information regarding the experimental setup can be found in the “Methods” section.

Our data assimilation procedure, named FastDA (Fast assimilation through DeepONet acceleration), adopts a pre-trained DeepONet, in lieu of the computationally demanding compressible Navier–Stokes equations. The DeepONet serves as a computationally efficient meta-model that establishes a connection between the oncoming flow disturbances input and the wall-pressure spectra output, as depicted in the assimilation loop in Fig. 1. Prior to deployment, the DeepONet is efficiently trained using supervised learning, with labeled data obtained from direct numerical simulations of the compressible Navier–Stokes equations, as illustrated in the training loop in the figure. Once trained, the same DeepONet is adopted for many sets of measurements, and we predict the oncoming disturbances associated with each set.

The major advantage of our proposed approach, relative to the previous ensemble-variational method^34^, is the significant reduction in computational expense. Including the initial training cost, we are able to perform approximately 2000 data-assimilation problems in the same wall-clock time that was required to solve just one inverse problem using EnVar. This cost reduction is primarily attributed to the application of the DeepONet as a meta-model for the compressible Navier–Stokes equations. Once trained, the DeepONet is three-orders-of-magnitude more efficient in terms of wall-clock time for each data assimilation solution.

The utilization of DeepONet requires that we address two notable challenges. The first is to optimize the selection of training data, and specifically to minimize the computational cost for data generation by expensive direct simulations of the Navier–Stokes equations. To address this issue, we efficiently parameterize the space of possible oncoming disturbances, model its statistics using a physics-based approach that exploits the available measurements, and use Latin hypercube sampling as a strategy to cover relevant ranges of parameters. The second challenge has to do with solving the inverse problem for a nonlinear and chaotic system which amplifies errors, in particular when the DeepONet introduces model errors. How do we navigate the tortuous optimization landscape as we search for the optimal oncoming disturbances that reproduce the measurements? For this task, we utilize an enhanced version of downhill simplex optimization, which exhibits improved performance for high-dimensional optimization problems.

## Problem formulation and results

Wall-measurements of transitional flows in high-speed flight tests and experiments are valuable. However, they are far from describing the rich and complex dynamics of the flow since the data are often collected by a limited number of localized sensors, and capture only one quantity rather than the entire flow state. The present study uses experimental measurements of wall pressure, which were described in detail by Kennedy et al.^10^. The main question that we address is the following: *‘given the wall-pressure spectra at a few probes, can we accurately and efficiently reconstruct the full flow field?’*

### Measurements and unknown inputs

The time series of wall pressure recordings from the experiments^10^ are processed to compute the spectra (Fig. 1) and to form the measurement vector,

1 $$\begin{aligned} \begin{aligned} \varvec{m} = [\log _{10}(|\hat{p}(s_1,f_1)|^2),\log _{10}(|\hat{p}(s_1,f_2)|^2),\dots , \log _{10}(|\hat{p}(s_{N_s},f_{N_f})|^2)] \end{aligned} \end{aligned}^{\top},$$

where $\hat{p}(s_{N_s},f_{N_f})$ is the wall-pressure spectral amplitude at sensor $s_i$ and frequency $f_j$, $N_s$ is the total number of sensors, and $N_f$ is the total number of discrete frequencies of interest. The measurements are initially assumed to be statistically stationary, and the entire 80-ms time series is used to compute the spectra using Welch’s method. This assumption enables us to compare to previous work, and is subsequently relaxed to demonstrate the efficiency and impact of our method. In all the data assimilation problems presented $\varvec{m} \in \mathbb {R}^{76\times 1}$ contains spectra with $f \in [50,75,...,500]$ kHz, $N_f = 19$, from four sensors, $N_s = 4$. Details on the computation of the spectra can be found in the “Methods” section.

In order to reconstruct the flow corresponding to these measurements, it is necessary to estimate the spectral makeup of the oncoming disturbances that amplify within the boundary layer, disturb the flow away from its laminar state, and ultimately lead to the recorded measurements. These disturbances are boundary-layer instability waves, which may be linear at their onset^16^, but quickly become nonlinear, interacting with one another and with the base state itself. The amplitudes of the individual instability waves thus have a profound impact on the flow evolution and, hence, on the sensor signals. Our objective is to identify the amplitudes of these waves, which together form our control vector $\varvec{c} \ge 0$ that we aim to optimize such that our computational predictions reproduce the measurements. Only then can we discover the experimental flow field.

We assume that the upstream flow, at the start of the domain of interest, is a linear superposition of a laminar state and fifty two instability waves with frequencies and integer azimuthal wavenumbers (*f*, *k*); details are provided in the “Methods” section. Thirteen frequencies are considered $f \in [50,75,...,350]$ kHz, and four integer azimuthal wavenumbers $k \in [0,20,40,60]$. The control vector is therefore a fifty-two dimensional, $\varvec{c} \in \mathbb {R}^{52\times 1}$, unknown input and searching for its optimal value can be challenging since the system is nonlinear and chaotic.

### Fast assimilation

Data assimilation is the procedure that attempts to minimize the difference between available experimental measurements $\varvec{m}$ and their computational prediction $\varvec{m}_{\scriptscriptstyle {S}}$. The latter depend on the amplitudes of the oncoming disturbances $\varvec{c}$ by the seemingly, and deceptively, simple expression $\varvec{m}_{\scriptscriptstyle {S}}= \mathcal {M}_{\scriptscriptstyle {S}}(\varvec{c})$, where $\mathcal {M}_{\scriptscriptstyle {S}}$ the combination of two steps. First, starting from $\varvec{c}$ the nonlinear, compressible, Navier–Stokes equations $\varvec{q} = \mathcal {N}(\varvec{c})$ predict the flow state $\varvec{q}$. Direct numerical simulation of this relation is considered the highest fidelity computational model. Second, measurements are extracted at the locations of the wall-pressure probes and the pressure spectra are evaluated. This step is encapsulated in the observation operator $\mathcal {H}$, where $\varvec{m}_{\scriptscriptstyle {S}}= \mathcal {H}(\varvec{q})=\mathcal {H}(\mathcal {N}(\varvec{c}))=\mathcal {M}_{\scriptscriptstyle {S}}(\varvec{c})$. The overall computational cost of these two steps is almost entirely due to the first one. When the computational model is direct numerical simulation, each evaluation $\mathcal {N}(\varvec{c})$ of the flow over the cone is a resource-intensive task, which requires 2880 CPU hours (5 wall-clock hours using 576 CPU cores) in the high performance computing cluster with Intel Xeon Gold Cascade Lake 6248R CPUs at Johns Hopkins University. This cost multiplies quickly because data assimilation involves an iterative minimum-seeking procedure where the estimate of $\varvec{c}$ is successively updated until the computational model outputs sufficiently approach the measurements.

The minimization is challenging in part due to the nonlinear nature of the compressible Navier–Stokes operator within $\mathcal {M}_{\scriptscriptstyle {S}}(\varvec{c})$, which requires an iterative optimization procedure. Iterative methods must grapple with the substantial computational demands of numerically solving the compressible Navier–Stokes equations at each iteration. This challenge is particularly pronounced when dealing with experiment- and flight-relevant Reynolds and Mach numbers, as is the case here with the measurements $\varvec{m}$. In addition, transitional flows are chaotic, and hence lead to an oscillatory cost landscape that is difficult to navigate.

**Figure 2:**
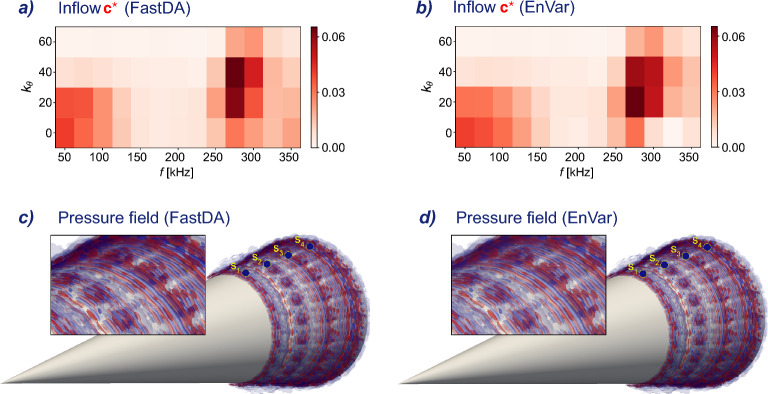
Comparison of inflows, the solution to the data assimilation problem, and the corresponding flow fields discovered with direct numerical simulations of the compressible Navier–Stokes equations. Left: FastDA (proposed method); right: EnVar (reference). Top: Inflow spectra $\varvec{c}$. Bottom: Pressure fluctuations iso-surfaces at $-0.1,-0.05,0.05,0.1$ (blue to red) at matching times.

The first, and to our knowledge only, instance in the literature, which attempts the assimilation of physical measurements $\varvec{m}$ from a hypersonic boundary-layer flow, is the recent work by Buchta et al.^34^. They were able to identify the optimal control vector $\varvec{c}_{\scriptscriptstyle {S}}^{\star }$ shown in Fig. 2, using an ensemble variational method (EnVar). EnVar addresses the nonlinear dependence of the cost function on the control parameters by adopting a local quadratic approximation, estimating the local gradient and Hessian using an ensemble of DNS evaluations, and iteratively repeating the gradient-descent procedure. The presence of chaotic dynamics, which leads to an oscillatory landscape of the cost function, is addressed with a number of countermeasures, e.g. attempting multiple initial guesses, inflation of the ensemble covariance matrix, randomizing the search directions. Each iteration of EnVar is computationally costly since it involves an ensemble of direct numerical simulations (DNS). Only the final estimated flow is retained and all the simulations that were performed prior to convergence are discarded. Should new experimental data be collected with a slightly different flow condition, the entire process must be repeated, thus incurring the same high computational cost. These properties render the method primarily suitable for application to a few high-valued experimental configurations rather than an efficient approach that can be widely adopted.

Here, we deploy a fast data assimilation algorithm (FastDA), which takes advantage of the capacity of a deep operator network (DeepONet) to learn nonlinear functionals. The DeepONet model $\mathcal {M}_{\scriptscriptstyle {D}}(\varvec{c})$ efficiently replaces the Navier–Stokes operator $\mathcal {M}_{\scriptscriptstyle {S}}(\varvec{c})$ and significantly reduces computational time to $\mathcal {O}(0.01)$ seconds per evaluation. FastDA comprises two steps. The first is the DeepONet training, involving minimal dataset generation, supervised learning, and storage of the trained network in memory. The second is the assimilation step, where the optimal control vector is computed through Bayesian estimation using the DeepONet and a gradient-free minimum-seeking technique.

The choice of a gradient-free method mitigates the effect of prediction errors introduced by a meta-model, which pose a challenge to gradient-based approaches due to the high sensitivity of derivatives to fluctuations in the function. Here, our meta-model is a DeepONet due to the desire to adopt an operator network that maps an input function, namely the inflow spectra $\varvec{c}$, to an output function, namely the wall-pressure spectra $\varvec{m}$. DeepOnets are specifically designed for this purpose^43,44^. These operator networks can be supplemented with governing equations in the training loss to make up for missing data in training^45^, in which case they are considered physics-informed DeepONets (PI-DeepONets). In the present application, our training data are complete since they are obtained from direct numerical simulations, and we focus on training a DeepONet that yields data-assimilation accuracy comparable to EnVar with DNS. We will demonstrate that our approach fulfills this requirement.

### Minimal dataset and training

The network is shown in Fig. 1, and is pre-trained before deployment as illustrated by the ‘DeepONet training’ loop, when the arrows are connected to $\textcircled {1}$.

Training data are generated using expensive DNS. Our goal is to minimize the number of samples to keep computational expenses low, while ensuring that data are both diverse and representative of probable realizations. To achieve this goal, we sample the subspace comprised of the most uncertain directions of the input. Specifically, we map $\varvec{c} \in \mathbb {R}^{52 \times 1}$ to a normal distribution, and reduce the dimension to the leading eigen-directions of the covariance matrix that collectively account for 95% of the total variance. The result is a ten-dimensional subspace, akin to a rank-ten dimensionality reduction by principal component analysis (PCA)^46^. This subspace is parameterized as $\varvec{c} = \phi (\varvec{w}_0 + \widetilde{\textbf{W}}\widetilde{\varvec{w}})$, where $\widetilde{\textbf{W}}\in \mathbb {R}^{52 \times 10}$ is the matrix of the ten eigen-directions and $\widetilde{\varvec{w}}\in \mathbb {R}^{10 \times 1}$ is sampled using LHS from a zero-mean normal distribution with diagonal covariance $\widetilde{\mathbf {\Lambda }}_{\varvec{w}}$ (see “Methods”).

A total of 68 samples were sufficient to limit the DeepONet’s prediction error, $\overline{\varepsilon }_{\scriptscriptstyle {D}}$, on validation data (not used in training) to 5% (mean-square-error, MSE). Therefore, we perform 68 DNS to gather the necessary measurements $\mathcal {M}_{\scriptscriptstyle {S}}(\varvec{c})$. To place this cost in perspective, EnVar required 110 evaluations of $\mathcal {N}$ for a single data assimilation problem. Moreover, to facilitate efficiency and accuracy of DeepONet training, we included a mapping from the 52-dimensional $\varvec{c}$ to the 10-dimensional $\widetilde{\varvec{w}}$ in the input layer of the DeepONet. This mapping can be motivated by the recent work by Kontolati et al.^47^ on the superior predictive accuracy of DeepONets when the inputs are mapped to reduced, or latent, spaces.

### Bayesian estimation

Once pre-trained, the DeepONet is deployed to predict the measurements $\mathcal {M}_{\scriptscriptstyle {D}}(\varvec{c})$, which facilitates swiftly searching the high-dimensional input space for the optimal amplitudes $\varvec{c}$ that reproduce the experimental measurements $\varvec{m}$ (the ‘Assimilation’ loop in Fig. 1, when the arrows are connected to $\textcircled {2}$).

We consider uncertain prior knowledge regarding $\varvec{c}$ represented by a likelihood function, $\mathcal {P}(\varvec{c})$, which is assumed to follow a log-normal distribution $log\mathscr {N}(\varvec{c}_0, \varvec{\Sigma }_{\varvec{c}})$. This choice implicitly enforces the constraint that the vector of amplitudes $\varvec{c}$ remains non-negative. Additionally, we introduce the transformation $\varvec{c} = \phi (\varvec{w})$ to represent the log-normal distribution using its corresponding normal distribution $\mathscr {N}(\varvec{w}_0,\varvec{\Sigma }_{\varvec{w}})$ and likelihood $\mathcal {P}(\varvec{w})$. Detailed information about this process is available in the “Methods” section.

Given experimental measurements $\varvec{m}$, we introduce the conditional likelihood $\mathcal {P}(\mathcal {M}_{\scriptscriptstyle {D}}(\varvec{w})| \varvec{w})$ with normal distribution $\mathscr {N}(\varvec{m},\varvec{\Sigma }_{\varvec{m}})$. With these likelihoods in place, we leverage Bayesian estimation to compute the optimal $\varvec{c}^{\star }=\phi (\varvec{w}^{\star })$ that best reconstructs $\varvec{m}$ as the maximizer of the likelihood $\mathcal {P}(\mathcal {M}_{\scriptscriptstyle {D}}(\varvec{w})| \varvec{w})\mathcal {P}(\varvec{w})$, or equivalently in the context of normal distributions as the minimizer of the negative log-likelihood,

2 $$\begin{aligned} \mathcal {J}_{\scriptscriptstyle {D}}(\varvec{w})= ||\varvec{m} - \mathcal {M}_{\scriptscriptstyle {D}}(\varvec{w})||^2_{\varvec{\Sigma }_{\varvec{m}}^{-1}} + || \varvec{w} - \varvec{w}_0||^2_{\varvec{\Sigma }_{\varvec{w}}^{-1}}. \end{aligned}$$

The second term in $\mathcal {J}_{\scriptscriptstyle {D}}(\varvec{w})$ can be interpreted as a regularization, and arises naturally in the Bayesian interpretation of the cost function as the negative log-likelihood of Bayes formula for a Gaussian distribution.

The conventional utilization of the Navier–Stokes operator $\mathcal {M}_{\scriptscriptstyle {S}}(\varvec{c})$ in place of the DeepONet model $\mathcal {M}_{\scriptscriptstyle {D}}(\varvec{c})$ results in a data assimilation problem involving the minimization of a different scalar function, $\mathcal {J}_{\scriptscriptstyle {S}}(\varvec{w})$. This distinction arises because the DeepONet $\mathcal {M}_{\scriptscriptstyle {D}}(\varvec{c})$ acts as a meta-model for $\mathcal {M}_{\scriptscriptstyle {S}}(\varvec{c})$, rendering $\mathcal {J}_{\scriptscriptstyle {D}}(\varvec{w})$ an approximation, or a noisy reproduction, of $\mathcal {J}_{\scriptscriptstyle {S}}(\varvec{w})$.

### Gradient-free minimum seeking

Minimizing $\mathcal {J}_{\scriptscriptstyle {D}}(\varvec{w})$ introduced an additional challenge relative to $\mathcal {J}_{\scriptscriptstyle {S}}(\varvec{w})$, since computing the gradient of the former amplifies errors in the approximation of the Navier–Stoke operator by the DeepONet. For this reason, we opt for gradient-free techniques which, while less efficient than gradient-based methods, offer greater robustness against false fluctuations in the cost landscape. We employ an advanced version of the Nelder-Mead simplex algorithm^48^, which is also effective at avoiding local minima and has improved convergence in high-dimensional search spaces. This algorithm can thus effectively navigate a cost function landscape that is tortuous, which is typical of chaotic dynamics as in our transitional boundary layer. Convergence is achieved with roughly 3000 evaluations of $\mathcal {M}_{\scriptscriptstyle {D}}$, equivalent to under 2 min on a standard one CPU desktop equipped with an Intel Xeon W-1250 CPU, and yields the inflow amplitudes $\varvec{c}_{\scriptscriptstyle {D}}^{\star }= \phi (\varvec{w}_{\scriptscriptstyle {D}}^{\star })$ based on

3 $$\begin{aligned} \varvec{w}_{\scriptscriptstyle {D}}^{\star }= \underset{\varvec{w}}{\arg \min } \mathcal {J}_{\scriptscriptstyle {D}}(\varvec{w}). \end{aligned}$$

The optimal control vector $\varvec{c}_{\scriptscriptstyle {D}}^{\star }$ may deviate from the inflow amplitudes $\varvec{c}_{\scriptscriptstyle {S}}^{\star }$ computed through the minimization of $\mathcal {J}_{\scriptscriptstyle {S}}(\varvec{w})$ due to the discussed differences between $\mathcal {J}_{\scriptscriptstyle {D}}(\varvec{w})$ and $\mathcal {J}_{\scriptscriptstyle {S}}(\varvec{w})$, arising from the discrepancy between $\mathcal {M}_{\scriptscriptstyle {S}}$ and $\mathcal {M}_{\scriptscriptstyle {D}}$. For our strategy to be successful, the deviation between the original solution involving $\mathcal {J}_{\scriptscriptstyle {S}}(\varvec{w})$ and our assimilation with FastDA needs to be accurate up to the validation error of the DeepONet. We now proceed to assess this aspect, and leave further details on dataset creation, neural network training and architecture, and the minimization process of $\mathcal {J}_{\scriptscriptstyle {D}}(\varvec{w})$ for the “Methods” section.

**Figure 3:**
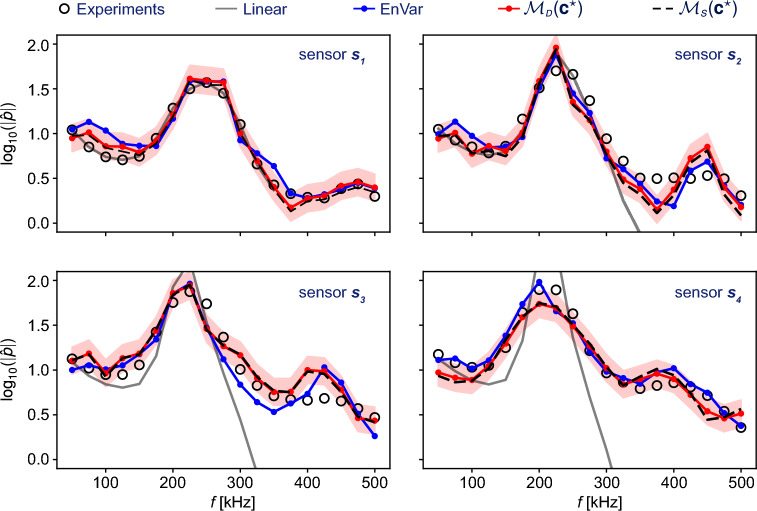
Pressure spectra at the four sensors $s_1-s_4$; measurements $\varvec{m}$ corresponding to different solutions $\varvec{c}^{\star }$ of the data assimilation problem. Circles: experimental data (99% confidence interval is $\log _{10}\left( |\hat{p}|\right) \pm 0.03$). Grey line: output $\varvec{m}$ corresponding to $\varvec{c}_{\scriptscriptstyle {\widetilde{\mathcal {N}}}}^{\star }$, explicit solution based on the linearized Navier–Stokes operator $\widetilde{\mathcal {N}}$. Blue line with dots: output $\mathcal {M}_{\scriptscriptstyle {S}}(\varvec{c}_{\scriptscriptstyle {S}}^{\star })$, EnVar solution (reference based solely on direct numerical simulations). Red solid line: output $\mathcal {M}_{\scriptscriptstyle {D}}(\varvec{c}_{\scriptscriptstyle {D}}^{\star })$, FastDA solution (proposed method). Red transparent band: ± three times the validation error of the DeepONet, $\mathcal {M}_{\scriptscriptstyle {D}}(\varvec{c}_{\scriptscriptstyle {D}}^{\star }) \pm 3\overline{\varepsilon }_{\scriptscriptstyle {D}}$. Black dashed line: output $\mathcal {M}_{\scriptscriptstyle {S}}(\varvec{c}_{\scriptscriptstyle {D}}^{\star })$, FastDA solution simulated with the Navier–Stokes operator $\mathcal {N}$.

### Results: accuracy and cost

Utilizing the same set of experimental measurements $\varvec{m}$ as in the recent study by Buchta et al.^34^, we solve the data assimilation problem by determining the inflow amplitudes through the minimization process described in Eq. (3). The corresponding inflow $\varvec{c}_{\scriptscriptstyle {D}}^{\star }= \phi (\varvec{w}_{\scriptscriptstyle {D}}^{\star })$ is visualized in Fig. 2. Notably, our results agree well with the recent predictions using the EnVar approach, which is based on direct numerical simulations of the Navier–Stokes equations as the forward model. This general agreement is evident when comparing the amplitude distribution across frequencies and azimuthal wave numbers (panels *a* and *b* of Fig. 2).

Similar to the EnVar prediction, the majority of the energy is distributed on three-dimensional waves rather than two-dimensional ones. This observation is important because it dispels an entrenched view that transition over cones is predominantly due to planar waves, which are the most exponentially unstable disturbances according to linear stability theory. However, this view is at odds with the measurements, which are most accurately reproduced by three-dimensional waves. One may remark that the importance of three-dimensionality should be expected since transition to turbulence requires it, but the extent to which early three-dimensional waves are relevant to the pre-transitional flow measurements was certainly underestimated prior to application of data assimilation to this problem.

The estimated inflow which is given by $\varvec{c}_{\scriptscriptstyle {D}}^{\star }$ is subsequently adopted in the simulation of the complete three-dimensional hypersonic boundary-layer flow, using direct computation of $\mathcal {N}(\varvec{c}_{\scriptscriptstyle {D}}^{\star })$. An instantaneous visualization of the field is shown in Fig. 2*d*, and for comparison the prediction from the EnVar approach is shown in panel *c*. The three-dimensional iso-surfaces show pressure fluctuations, and agreement between the two figures is evident. The flow exhibits the anticipated streamwise and spanwise fluctuations. In effect, this type of result is the most valuable outcome of solving the inverse problem, since we can now evaluate any flow quantity of interest at full resolution, when the measurements were limited to a few pressure sensors along the wall.

The fidelity of our predictions in reproducing the measurements is important, and is examined in Fig. 3. The figure compares the available experimental data $\varvec{m}$, their reproduction using DeepONet $\mathcal {M}_{\scriptscriptstyle {D}}(\varvec{c}^{\star })$, and their reproduction by Navier–Stokes simulation of the DeepONet-predicted inflow $\mathcal {M}_{\scriptscriptstyle {S}}(\varvec{c}^{\star })$. For reference, the figure also shows two limiting cases: the prediction if a linear model is assumed for the forward dynamics and the prediction from EnVar. Notably, the DeepONet effectively captures the primary and secondary peaks in the spectra, which correspond to the primary instability waves and the nonlinear generation of the harmonic. While the primary wave has its origin in linear stability theory, predicting its correct amplitude downstream requires an accurate nonlinear forward model. This point is evident by sensors 3 and 4, where the linear model deviates appreciably from the experiments. In contrast, the DeepONet-dependent results $\mathcal {M}_{\scriptscriptstyle {D}}(\varvec{c}^{\star })$ and $\mathcal {M}_{\scriptscriptstyle {S}}(\varvec{c}^{\star })$ accurately reproduce the measurements, within the validation error of the network and as accurate as EnVar. The prediction of the secondary peak due to the nonlinear generation of harmonic frequencies is also within the same level of accuracy as EnVar (note that the figure is in logarithmic scale). To quantify the discrepancies between these curves, we compute the normalized error,

4 $$\begin{aligned} \delta = {||\varvec{m} - \mathcal {M}(\varvec{c})||_{\varvec{\Sigma }_{\varvec{m}}^{-1}} \over ||\varvec{m}||_{\varvec{\Sigma }_{\varvec{m}}^{-1}}}. \end{aligned}$$

The results reveal small differences in the accuracy of EnVar, $\delta (\mathcal {M}_{\scriptscriptstyle {S}}(\varvec{c}_{\scriptscriptstyle {S}}^{\star })) = 9.98\%$, compared to the DeepONet outcomes, $\delta (\mathcal {M}_{\scriptscriptstyle {D}}(\varvec{c}_{\scriptscriptstyle {D}}^{\star })) = 10.67\%$ and $\delta (\mathcal {M}_{\scriptscriptstyle {S}}(\varvec{c}_{\scriptscriptstyle {D}}^{\star }))=10.83\%$.

In effect, the reproduction of the measurements is similar in accuracy with either the EnVar or FastDA. This agreement and the accuracy of the predicted state confirm that deploying DeepONet as an approximation of the compressible Navier–Stokes equation is an effective and, importantly, efficient approach to accelerate data assimilation. Additionally, the limitations in achieving a more accurate assimilated flow does not appear to hinge on improving the DeepONet model which in our FastDA yields $\delta (\mathcal {M}_{\scriptscriptstyle {S}}(\varvec{c}_{\scriptscriptstyle {D}}^{\star }))=10.83\%$. In the limit of a perfect DeepONet prediction, we recover the Navier–Stokes operator which in EnVar led to $\delta (\mathcal {M}_{\scriptscriptstyle {S}}(\varvec{c}_{\scriptscriptstyle {S}}^{\star })) = 9.98\%$. As such, a more accurate meta-model, although it may reduce the prediction errors, would not lead to substantial improvement in the accuracy of solving the assimilation problem. Achieving a more accurate data-assimilation solution may require a different strategy. Given the dimensionality of the problem, the most effective approach is likely to increase the number of sensors, both in the streamwise and azimuthal directions. Such increase would in fact benefit any assimilation method. Exploring this avenue is, however, beyond the scope of our effort which focuses primarily on expediting the data assimilation process, while preserving accuracy against the most recent advances in the field. Our results demonstrate that we have achieved the accuracy goal, and we now comment on the efficiency of our approach.

**Figure 4:**
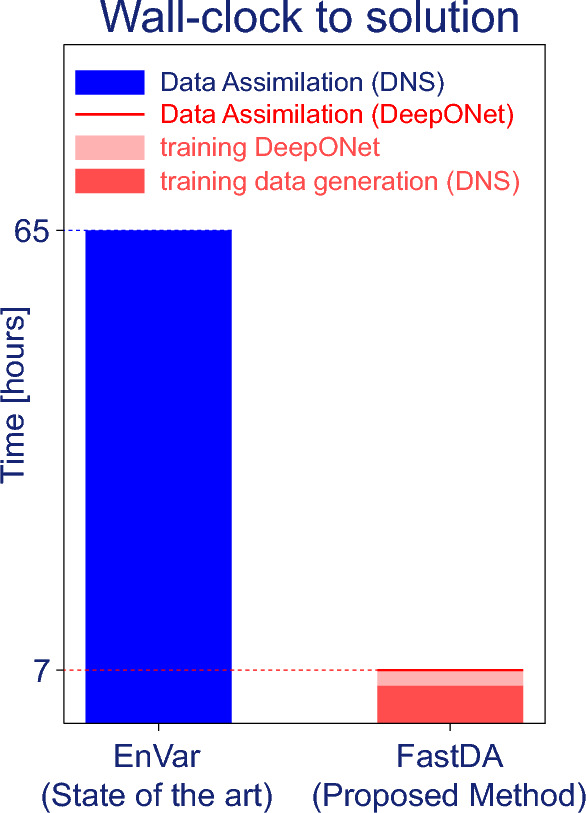
Wall-clock time to solution for the same DA problem. EnVar: 65 wall-clock hours (316, 800 CPU hours) on a HPC cluster with Intel Xeon Gold Cascade Lake 6248R CPUs (Data Assimilation). FastDA: 5 wall-clock hours (195, 840 CPU hours) on same HPC cluster (generation of training data), 2 GPU hours on one NVIDIA RTX A4000 GPU (DeepONet training), 1.73 CPU minutes on one Intel Xeon W-1250 CPU (Data Assimilation).

In terms of computational cost, our FastDA takes a very small fraction of the wall-clock time requirements of EnVar to solve the same data-assimilation problem. The computational cost of both approaches is reported in Fig. 4, in wall-clock time to emphasize the time to solution independent of the number of computational cores used in the algorithm. Using this metric, FastDA far outperforms EnVar, even if we include the time for the generation of the training data and for training. If we consider the CPU time, the difference is more striking. The wall-clock time of the EnVar algorithm corresponds to 316, 800 CPU hours (110 DNS, 2, 880 CPU hours each), since members of each ensemble are simulated simultaneously and each of them employs many processors. For FastDA, the offline one-time generation of the training data with DNS required the majority of the time, 195, 840 CPU hours (68 DNS, 2, 880 CPU hours each), and an additional two GPU hours were required for training the network. The live data-assimilation itself, which is the search for the optimal control vector that minimizes $\mathcal {J}_{\scriptscriptstyle {D}}(\varvec{w})$, was performed in 1.73 min on one CPU. In addition, the pre-trained DeepONet can be re-used to solve new data-assimilation problems associated with new experimental measurements $\varvec{m}$, each within the same 2-min timescale. Specifically, we can estimate the oncoming disturbance spectra $\varvec{c}$ for approximately 2, 000 additional experimental measurements $\varvec{m}$ on a single CPU in the same wall-clock time required for EnVar to solve one data assimilation problem on 576 CPUs (Fig. 4). We now provide examples of the additional data assimilations that we performed.

**Figure 5:**
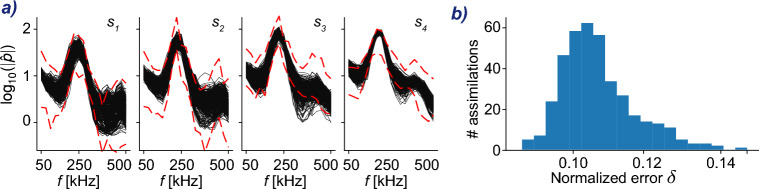
Left (**a**): cohort of $392$ experimental measurements (black solid lines); boundaries of training dataset (red dashed lines). Right (**b**): performance of fast data assimilation with FastDA on the cohort of $392$ experimental measurements; each bar spans a 0.3581% interval of discrepancy error $\delta$ (Eq. 4); each column represents the amount of different data assimilation problems within an interval of error values; discrepancy error computed as in Eq. (4).

**Figure 6:**
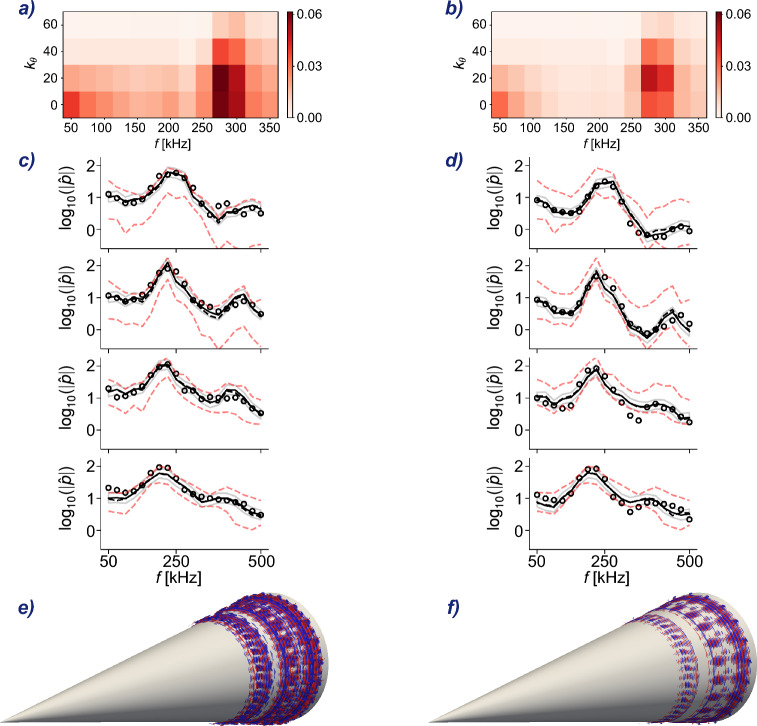
Assimilation with FastDA of two from the 392 cases in Fig. 5. Left column: $\delta (\mathcal {M}_{\scriptscriptstyle {D}}(\varvec{c}_{\scriptscriptstyle {D}}^{\star })) = 9.65\%$; right column: $\delta (\mathcal {M}_{\scriptscriptstyle {D}}(\varvec{c}_{\scriptscriptstyle {D}}^{\star })) = 9.87\%$. (*a,b*): Inflows $\varvec{c}_{\scriptscriptstyle {D}}^{\star }$. (*c,d*): Experimental measurements (open black circles, with 99% confidence intervals $\log _{10}\left( |\hat{p}|\right) \pm 0.11$ and $\pm 0.07)$, DeepONet prediction $\mathcal {M}_{\scriptscriptstyle {D}}(\varvec{c}_{\scriptscriptstyle {D}}^{\star })$ (black solid line), ± three times the validation error of the DeepONet, $\mathcal {M}_{\scriptscriptstyle {D}}(\varvec{c}_{\scriptscriptstyle {D}}^{\star }) \pm 3\overline{\varepsilon }_{\scriptscriptstyle {D}}$ (grey transparent band), DNS prediction from FastDA-estimated control vector $\mathcal {M}_{\scriptscriptstyle {S}}(\varvec{c}_{\scriptscriptstyle {D}}^{\star })$ (black dashed line), boundaries of the training dataset (red dashed lines). (*e, f*): Pressure fluctuations iso-surfaces at $-0.1,-0.05,0.05,0.1$ (blue to red) from DNS of FastDA-estimated $\varvec{c}_{\scriptscriptstyle {D}}^{\star }$.

### FastDA on a cohort of measurements

The time advantage achieved with our FastDA enables efficient solution of a large number of data-assimilation problems, which is impractical using conventional methods. For example, due to the cost of EnVar, Buchta et al^34^ used the entire 80 ms time series from the PCB sensors to compute the wall-pressure spectra. Their implicit assumption is that the experimental data is statistically stationary during this duration. Dividing the time series into shorter horizons and assimilating the spectra associated with different sub-intervals was not conceivable given the associated computational cost. Our FastDA approach enables us to consider the many separate cases that result from dividing the 80 ms time series into hundreds of locally stationary segments. We extracted $392$ such intervals, applied Welch’s method within each of these segments, computed the associated $392$ wall-pressure spectra $\varvec{m}$, and assimilated each of them using FastDA. Our approach thus unlocks the possibility to perform a comprehensive study of inflow variability during the experiment.

The $392$ wall-pressure spectra $\varvec{m}$ are illustrated in Fig. 5 together with the boundaries of the training data. The orders-of-magnitude variability of the spectra $\varvec{m}$ shown in the figure demonstrates that the inflow $\varvec{c}$ may actually not be stationary, which further underscores the need for a fast data assimilation approach. Moreover, it is noteworthy that, in the frequency range $f=[50,350]$ kHz, the spectra of the first sensor are perfectly contained within the training dataset. This demonstrates the efficacy of the design of our training dataset with sufficient variance.

The discrepancy $\delta (\mathcal {M}_{\scriptscriptstyle {D}}(\varvec{c}_{{\scriptscriptstyle {D}},i}^{\star }))$ measured according to Eq. (4) for each of the $i=\{1,\dots , 392\}$ newly solved data-assimilation problems is presented in Fig. 5b. The predicted control vectors using FastDA reproduce the measurements with accuracy on the order of $10\%$ and with mild variability, similar to the earlier results. The similarity indicates that our method is both effective and robust in performance for measurements within the physics-based design of the training space. The wall-clock time to solution for the complete set of $392$ problems is approximately 11 h on a desktop with a NVIDIA RTX A4000 GPU, 32GB of RAM, and an Intel Xeon W-1250 CPU, with solutions computed in series each requiring less than 2 min.

Two solutions from the set of data-assimilation problems are presented in detail in Fig. 6. The inflows $\varvec{c}$ in panels (*a*) and (*b*) present a considerable difference in the amplitudes of all frequency and wave-number pairs, especially the most energetic pairs. The range of these energetic waves is the same in both cases. However, since transitional flows are extremely sensitive to variations of the inflow conditions, differences are amplified downstream and the pressure fluctuations at the sensors deviate by orders of magnitude (panels (*c*) and (*d*)). A comparison of the respective pressure fields is shown in panels (*e*) and (*f*), which highlights the qualitative differences in the two states of the flow, during the same experiment.

These appreciable changes in the flow state are important in the study of hypersonic flows, and are not possible to discern if the entire measurement horizon is assumed to be statistically stationary in a single data assimilation. Our FastDA enables us to assimilate valuable measurements in much finer detail, previously not possible due to computational cost of conventional methods. The present results thus demonstrate the potential role of deep learning in data assimilation for nonlinear, transitional, hypersonic flows.

## Conclusions

We have shown how deep learning can be leveraged to drastically accelerate the solution of a data-assimilation problem based on a realistic scenario. The configuration is a Mach 6, transitional, boundary-layer flow over a cone with half-angle $7^{\circ }$. Only one previous study has successfully performed assimilation of such experimental measurements^34^, using an ensemble-variational method that is computationally costly. Since the majority of the computational cost is associated with performing high-fidelity simulations of the compressible Navier–Stokes equations, we put forward the idea to replace the original high-fidelity model with a computationally efficient deep operator network (DeepONet) previously trained on far fewer high-fidelity simulations.

The proposed approach required us to tackle two key challenges. The first is the design of the training data, which are expensive to compute using high-fidelity simulations, must span a 52 dimensional input space, and ensure that the corresponding output space spans potential measurements of interest. We overcame this challenge by reducing the input space to the most uncertain search directions, using Latin hypercube sampling to span the inputs, and by adopting a physics-based design of the distribution of samples to span the output space of interest. The second challenge is to solve the data-assimilation minimization problem when the cost-function landscape is affected by the unknown errors of the DeepONet. We facilitated the training of the network by mapping the input layer to the reduced search space, effectively mimicking the so-called L-DeepONet^47^, and demonstrated the capability of an accelerated gradient-free minimization method to find the solution $\varvec{c}$ of the data-assimilation process.

The trained low-cost DeepONet is then deployed to solve the data-assimilation problem. Starting from the experimental wall-pressure spectra at the sensors, our FastDA accurately predicts the amplitudes of the oncoming disturbances, in comparison to the only available data-assimilation result in the literature. The computational cost is significantly reduced, from tens of hours to minutes of wall-clock time (from 65 h to 1.73 min). The time savings allow us to solve many new data-assimilation problems, efficiently. As such, we examined the experimental measurement in fine detail, by dividing the measurement horizon into segments and assimilating each of them independently. The results demonstrated the significant variability in the flow state during the experiment, as well as the robustness and efficiency of our FastDA approach.

Finally, we illustrated the determination of full 3D flow fields from the discovered inflow condition for some select cases by visualizing iso-pressure surfaces. These fields are consistent with the surface pressure data.

Future efforts can integrate uncertainty quantification into the deep-learning framework to provide estimates of prediction uncertainty. This step could be particularly helpful when the training data are contaminated with noise, for example if they are sourced from experiments or lower fidelity computations. Additionally, optimizing sensor placement can enhance the accuracy of data assimilation, especially in situations with limited experimental measurements. Leveraging the DeepONet model can accelerate this process by efficiently mapping inflow spectra to potential sensor placements downstream.

## Methods

### Experimental setup

The experimental measurements used in this work were detailed in the work by Kennedy et al.^10^. The experiment was performed at the Air Force Research Laboratory, in a Mach 6 Ludwieg tube facility [see Kimmel et al.^49^ for details]. When a fast-acting valve is opened, compressed and heated gas from the driver section of the tube accelerates through the converging-diverging nozzle. The free-stream conditions in the test section are $U_{\infty } = 904$ m s^-1^, $T_{\infty }=54$ K, and $\rho _{\infty }=0.0274$ kg m^-3^, and the associated per-meter Reynolds number is $Re_{\infty }/L=7.11 \times 10^6$ m^-1^. Each tunnel firing yields two intervals of measurements, each 100 ms, that are assumed to be stationary. The data we used are 80 ms within the second interval.

The cone cross-section is circular, its half-angle is $7^{\circ }$, its length is 414 mm, and its leading-edge has radius $r_{n} = 0.508$ mm which is considered sharp. At the start of the experiment, the cone is at room temperature, which is essentially maintained during the short-duration test. The experiment was performed at nominally zero angle of attack, and wall-pressure measurements were recorded at 5 MHz using six PCB model 132A pressure sensors. Four of the sensors were placed along a streamwise ray from the leading edge, at positions $(s_1,s_2,s_3,s_4)=(215,241,266,291,316)$ mm. The fourth sensor was flanked in the azimuthal direction by two sensors, each displaced by $\pm 8.5^{\circ }$.

### Wall-pressure spectra of experimental measurements

An 80 ms recording of the wall-pressure was used from each sensor data to compute the wall-pressure spectra $\varvec{m}$ that are the measurements for the data assimilation. The evaluation of the spectra was performed using Welch method^50^, with non-overlapping, Hann-windowed bins of 0.08 ms. These parameters were unchanged throughout the study.

Earlier published papers used the entire 80 ms to compute the spectra^10,34^, effectively assuming statistical stationarity for the entire duration. Here, we have additionally computed the wall-pressure spectra from shorter intervals of the entire 80 ms time-trace, and assimilated these measurements. Specifically, we computed the spectra from intervals $[t_0,t_0 + T_l]$, with $T_l = \{4,6,8,\dots ,80\}$ ms. The wall-pressure spectra computed from the shortest interval $T_l=4$ ms contains 50 bins, and that from the longest interval $T_l=80$ ms contains 1000 bins. For a given $T_l$, we can obtain multiple spectra by shifting $t_0$ which we have varied with an increment $0.25T_l$. Using this approach, the total number of wall-pressure spectra that we can extract from the time series is $N_{exp} = 392$, each of which can be assimilated to predict the flow within the associated time interval within the experiment.

For each spectra that we adopt as measurements, we quantify the uncertainty of the Welch estimate using the 99% confidence interval of a $\chi ^2$ distribution^50^. This confidence interval varies with the number of bins. When only 50 bins are available, the interval is $\log _{10}\left( |\hat{p}|\right) \pm 0.15$, and reduces to $\log _{10}\left( |\hat{p}|\right) \pm 0.03$ when there are 1000 bins. These values should be viewed against the magnitude of $\log _{10}\left( |\hat{p}|\right)$, which is order one.

### Compressible Navier–Stokes

The flow considered in this work satisfies the compressible Navier–Stokes equations. We adopt as reference scales the free-stream fluid properties (density $\rho ^{*}_{\infty }$, shear viscosity $\mu ^{*}_{\infty }$, specific heat at constant pressure $C^{*}_{p,\infty }$), the free-stream speed of sound $c^{*}_{\infty }$, and the length $L^{*}=1$ m. Using these reference quantities, the non-dimensional Navier–Stokes equations are,

5 $$\begin{aligned}&\frac{\partial \rho }{\partial t} + \frac{\partial (\rho u_j)}{\partial x_j} = 0, \end{aligned}$$

6 $$\begin{aligned}&\frac{\partial \rho u_i}{\partial t} + \frac{\partial }{\partial x_j}(\rho u_i u_j) = - \frac{\partial p}{\partial x_i} + \frac{\partial \tau _{ij}}{\partial x_j},\end{aligned}$$

7 $$\begin{aligned} \frac{\partial E}{\partial t} + \frac{\partial }{\partial x_j} [u_j(E+p)] & = \frac{\partial }{\partial x_j} (u_i \tau _{ij}) + \frac{\partial }{\partial x_j} \left( \frac{\mu }{Re_c Pr}\frac{\partial T}{\partial x_j}\right) . \end{aligned}$$

The velocity vector in the *i*-direction is $u_i$, the pressure is *p*, the total energy is $E = p/(\gamma -1)+0.5\rho u_i u_i$, the temperature is $T=(\gamma p/\rho )/(\gamma -1)$, and $\gamma$ is the ratio of specific heats. The viscous stress tensor is defined as,

8 $$\begin{aligned} \tau _{ij} = \frac{\mu }{Re_c} \left( \frac{\partial u_i}{\partial x_j} + \frac{\partial u_j}{\partial x_i} \right) + \frac{3\mu _b - 2\mu }{3 Re_c} \delta _{ij} \frac{\partial u_k}{\partial x_k}, \end{aligned}$$

where $\mu _b$ is the bulk viscosity, and the dependence of the shear viscosity on temperature is given by $\mu = [(\gamma -1)T]^{n}$. The Reynolds and Prandtl numbers are $Re_c=\rho _{\infty }^{*}c_{\infty }^{*}L^{*}/\mu _{\infty }^{*}$ and $Pr = C_{p,\infty }^{*} \mu _{\infty }^{*}/k^{*}$, where $k^*$ is the conductivity. In compact operator notation, the governing equations are expressed as $\varvec{q} = \mathcal {N}(\varvec{c})$, where $\varvec{c}$ are the simulation parameters (e.g. inflow disturbance amplitudes) and $\varvec{q} = [\rho ,\rho \varvec{u},E]^{\top }$ is the flow state. Observations are extracted from the state according to $\varvec{m}_{\scriptscriptstyle {S}}= \mathcal {H}(\varvec{q}) = \mathcal {H}(\mathcal {N}(\varvec{c}))=\mathcal {M}_{\scriptscriptstyle {S}}(\varvec{c})$, where $\varvec{m}_{\scriptscriptstyle {S}}$ are the wall-pressure spectra from probes located on the wall as in the experimental setup. The high-fidelity simulations that generate the training data solve Eqs. (5–7) downstream of the cone leading-edge shock (see e.g. Fig 1), and are designed to accurately model the experimental configuration.

The inflow Reynolds number based on the post-shock, boundary-layer, edge conditions ($U_e=880\,\text {m/s}$, $T_e=65\,\text {K}$, and $\rho _\infty =0.0458\,\text {kg/m}^3$) is $Re_o\equiv \rho _e U_e L_o/\mu _e =1{,}345$ using the length scale $L_o = \sqrt{\mu _e \xi _o / \rho _e U_e}$. The flow at the inlet to the computational domain is a superposition of an equilibrium (steady) base state $\varvec{q}_{\scriptscriptstyle {B}}$ and instability waves $\sum _{\scriptscriptstyle {n,m}} c_{\scriptscriptstyle {{n,m}}} \widehat{\varvec{q}}_{\scriptscriptstyle {{n,m}}}$, where $c_{\scriptscriptstyle {{n,m}}}$ are the amplitudes and $\widehat{\varvec{q}}_{\scriptscriptstyle {{n,m}}}$ are the discrete, slow-mode linear instability waves at the frequency and integer azimuthal wavenumber pair ($\omega _n$, $k_m$).

We consider 52 pairs, with frequencies $f\in \left[ 50,75,\ldots ,350\right] \,\text {kHz}$ and integer azimuthal wavenumbers $k\in \left[ 0,20,40,60\right]$. The data assimilation searches for the vector $\textbf{c}=[\ldots ,c_{\scriptscriptstyle {{n,m}}},\ldots ]^\top$ of modal amplitudes at the inlet, which accurately reproduces available measurements.

DNS are performed using the algorithm developed and described in detail by Vishnampet et al.^51^, in a computational domain with sizes $(L_\xi , L_\eta ,L_\phi )$=(105 mm, 17.6 mm, $36^\circ$). This domain is discretized using $(N_s, N_y, N_\theta )=(751, 201, 108)$ grid points. linearized Navier–Stokes about $\varvec{q}_{\scriptscriptstyle {B}}$

### The distribution of the input space

The distribution of $\varvec{c} \in \mathbb {R}^{52 \times 1}$ is $log\mathscr {N}(\varvec{c}_0,\varvec{\Sigma }_{\varvec{c}})$, with corresponding normal distribution $\mathscr {N}(\varvec{w}_0,\varvec{\Sigma }_{\varvec{w}})$. Here, $\varvec{\Sigma }_{\varvec{c}}\in \mathbb {R}^{52 \times 52}$, $\varvec{w}_0 \in \mathbb {R}^{52 \times 1}$, and $\varvec{\Sigma }_{\varvec{w}}\in \mathbb {R}^{52 \times 52}$. The following element-wise relationships for the means and covariances hold,

9 $$\begin{aligned} c_{0,i} = e^{w_{0,i}+\frac{1}{2} \Sigma _{w,ii}}, \end{aligned}$$

10 $$\begin{aligned} \Sigma _{c,ij} = e^{w_{0,i}+w_{0,j} + \frac{1}{2}(\Sigma _{w,ii}+\Sigma _{w,jj})} \left( e^{\Sigma _{w,ij}} -1 \right) , \end{aligned}$$

with $i=1,\dots ,52$ and $j=1,\dots ,52$. Each element of the vector $\varvec{c}$ can be expressed as

11 $$\begin{aligned} c_i = e^{w_i}, \end{aligned}$$

which defines the one-to-one mapping $\varvec{c} = \phi (\varvec{w})$.

Buchta et al.^34^ generated their initial estimate of $\varvec{c}_0 = \varvec{c}_{\scriptscriptstyle {\widetilde{\mathcal{N}}}}^{\star }$ using a physics-based approach, which amounts to using the linearized Navier–Stokes operator in the cost function $\mathcal {J}_{\scriptscriptstyle {S}}(\varvec{w})$ and performing a one-shot, least-squares solution. For the nonlinear optimization, they reduced the dimensionality of the search space by focusing only on the subspace where the input has the majority of uncertainty, which corresponds to the space spanned by the ten leading eigenfunctions of their ensemble covariance $\varvec{\Sigma }_{\varvec{c}}$.

We search the same sub-space: We select $\varvec{c}_0 = \varvec{c}_{\scriptscriptstyle {\widetilde{\mathcal{N}}}}^{\star }$ and the original $\varvec{\Sigma }_{\varvec{c}}$ as the mean and covariance of the distribution in the input, control-vector space, which we map using $\varvec{c} = \phi (\varvec{w})$, or more specifically Eqs. (9)-(10), to $\varvec{w}_0$ and $\varvec{\Sigma }_{\varvec{w}}$. We perform an eigen-decomposition $\varvec{\Sigma }_{\varvec{w}}= \textbf{W}\mathbf {\Lambda }_{\varvec{w}}\textbf{W}^{\top }$, and write $\varvec{w} = \varvec{w}_0 + \widetilde{\textbf{W}}\widetilde{\varvec{w}}$ where $\widetilde{\textbf{W}}\in \mathbb {R}^{52 \times 10}$ are the first ten leading eigenvectors of $\varvec{\Sigma }_{\varvec{w}}$ and $\widetilde{\varvec{w}}\in \mathbb {R}^{10 \times 1}$ normally distributed $\mathscr {N}(\varvec{0},\widetilde{\mathbf {\Lambda }}_{\varvec{w}})$ with zero mean and covariance $\widetilde{\mathbf {\Lambda }}_{\varvec{w}}\in \mathbb {R}^{10 \times 10}$.

Retaining these ten eigen-directions is equivalent to a rank-ten dimensionality reduction through principal component analysis (PCA), or in the continuous form Karhunen-Loève (KLD) or proper orthogonal (POD) decomposition^46^. The eigenvalues associated with these ten leading directions satisfy,

12 $$\begin{aligned} \sum _{i=1}^{10} eig(\varvec{\Sigma }_{\varvec{w}})_i > 0.95 \sum _{i=1}^{52} eig(\varvec{\Sigma }_{\varvec{w}})_i, \end{aligned}$$

which is over 95% accuracy in approximating the covariance matrix. Since the eigenvalues represent the variances along the eigen-directions, this level of accuracy implies that samples of the control vector from this sub-space explore the major uncertainty.

We inflate the rank-ten covariance, $\widetilde{\varvec{\Sigma }}_{\varvec{w}}$, by a factor of one hundred. This factor was determined using a physics-based approach. The variance of the input distribution is designed such that the training data would likely span an ensemble of available experimental measurements in the output space. This step would normally be computationally costly because statistics of the measurements are nonlinear functions of those of the inputs, due to the nonlinearity of the Navier–Stokes equations. To mitigate this computational cost, we adopt the linearized operator $\widetilde{\mathcal {N}}$, and only consider the first sensor location. As previously illustrated in Fig. 3, the linear predictions are accurate at this most upstream sensor, $s_1$. We subsequently verified that the choice is adequate, and that the training data satisfactorily samples the output space of the measurements, from all the sensors and in the full nonlinear problem. Finally, we sample $\mathscr {N}(\varvec{w}_0,\widetilde{\varvec{\Sigma }}_{\varvec{w}})$, which has an associated log-normal distribution $log\mathscr {N}(\varvec{c}_0,\widetilde{\varvec{\Sigma }}_{\varvec{c}})$.

**Figure 7:**
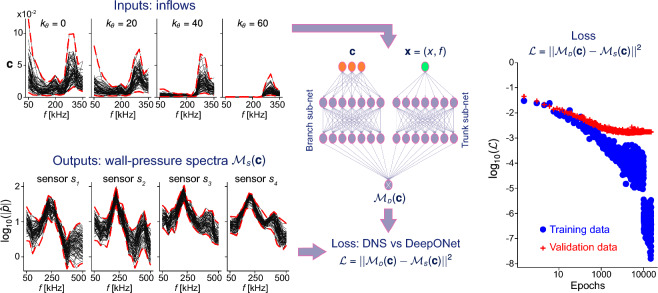
Left: training data obtained via DNS, with 68 inflows chosen with Latin Hypercube Sampling on the prior distribution of $\varvec{c}$. Center: DeepONet architecture: branch and trunk sub-networks with 10 layers each and 60 neurons per layer. Right: data loss $\mathcal {L}$ for the training data (blue) and the validation data (red).

### Latin hypercube sampling

In order to generate the training data, we sample the input distribution using Latin hypercube sampling (LHS). Since LHS is built to sample points in a hypercube, which correspond to sampling multivariate uniform distributions, we apply LHS to sample a uniform distribution $\mathcal {U}(\varvec{0},\textbf{I})$ with mean $\varvec{0} \in \mathbb {R}^{10 \times 1}$, covariance $\textbf{I} \in \mathbb {R}^{10 \times 10}$, and each element of the random vector defined on the interval [0, 1]. The samples are then mapped to be samples $\varvec{z} \in \mathbb {R}^{10 \times 1}$ of the normal distribution $\mathscr {N}(\varvec{0},\textbf{I})$ by using the inverse of the cumulative distribution function (CDF). Note, the CDF of independent identically distributed (normal) random variables is the product of the CDF of each random variable, and the CDF is bound in the interval [0, 1]. Therefore, the inverse of the CDF can be applied to each element of the random vector separately. The samples of the $\mathscr {N}(\varvec{0},\textbf{I})$ are then mapped to be samples of $\mathscr {N}(\varvec{w}_0,\widetilde{\varvec{\Sigma }}_{\varvec{w}})$ with the linear transformation $\varvec{w} = \varvec{w}_0 + \widetilde{\textbf{W}}\widetilde{\mathbf {\Lambda }}_{\varvec{w}}^{1/2}\varvec{z}$, which ultimately lead to samples from the desired log-normal $log\mathscr {N}(\varvec{c}_0,\widetilde{\varvec{\Sigma }}_{\varvec{c}})$.

To determine the optimal number of samples, we adopt the discrepancy criterion^52^, which is a uniformity criterion that assesses the space filling by a number of samples in a hypercube. The discrepancy can also be interpreted as a measure of the distance between the analytic cumulative distribution function and its sample-based counterpart. Here, we sample 68 different $\varvec{c}=\phi (\widetilde{\varvec{w}})$, corresponding to a discrepancy of approximately $10\%$. This number can be put in context by noting that EnVar required a total of 110 evaluations of $\mathcal {N}$ to solve a single data-assimilation problem.

### Learning the parameter space

Supervised learning is employed to train the DeepONet. The network models a nonlinear functional that maps the amplitudes of the oncoming disturbance amplitudes $\varvec{c}$ to the wall-pressure spectra $\mathcal {M}_{\scriptscriptstyle {D}}(\varvec{c})(\varvec{x})$, where $\varvec{x}=(x_i,f_j)$ includes the sensor position and the frequency of interest. The network output is selected to directly predict the wall-spectra at four locations, rather than predicting the entire three-dimensional flow field, with the purpose of solving the data assimilation problem using the available wall-mounted sensor data. The DeepONet architecture is comprised of branch and trunk networks (see Fig. 7), each with 10 layers and 60 neurons per layer. Every neuron but the output layer of the branch network include an ELU activation function. The input to the branch is the vector of oncoming disturbance amplitudes $\varvec{c}$, and the input to the trunk is the downstream location and frequency $\varvec{x}=(x_i,f_j)$ where we wish to predict the measurement. The output to the DeepONet is a dot-product of the outputs of the branch and trunk networks. The final outcome is therefore a weighted superposition of a learned basis, where the weights are the output of the branch (and hence the output layer of the branch does not include activation function) and the basis is the output of the trunk.

Since the equations that govern the nonlinear operator are known, namely the compressible Navier–Stokes equations, a set of labelled data

13 $$\begin{aligned} \begin{aligned} \mathfrak {D} = \{(\varvec{c}_1,\mathcal {M}_{\scriptscriptstyle {S}}(\varvec{c}_1)(x_1,f_1)),(\varvec{c}_1,\mathcal {M}_{\scriptscriptstyle {S}}(\varvec{c}_1)(x_1,f_2), \dots ,(\varvec{c}_2,\mathcal {M}_{\scriptscriptstyle {S}}(\varvec{c}_2)(x_1,f_1),\dots \} \end{aligned} \end{aligned}$$

is generated using direct numerical simulations. This dataset is used for supervised learning with a loss function:

14 $$\begin{aligned} \mathcal {L}_{\scriptscriptstyle {T}} = \mathcal {L}+ \mathcal {L}_{\scriptscriptstyle {WD}}. \end{aligned}$$

The first term represents the error between DeepONet predictions and training data,

15 $$\begin{aligned} \mathcal {L}= \frac{1}{N_{\mathfrak {D}}} \sum _{\varvec{c},\varvec{x}} \left| \mathcal {M}_{\scriptscriptstyle {D}}(\varvec{c})(\varvec{x}) - \mathcal {M}_{\scriptscriptstyle {S}}(\varvec{c})(\varvec{x}) \right| ^2 \end{aligned}$$

with $N_{\mathfrak {D}}$ is the cardinality of the dataset. The second term, $\mathcal {L}_{\scriptscriptstyle {WD}}$, is the typical *weight decay* regularization term^53^. The optimization is performed with the Adam optimizer^54^. The learning rate is $5\times 10^{-4}$ for the first 10,000 epochs and is changed to $5\times 10^{-5}$ for the remainder 5000 epochs.

Figure 7 presents the structure of the dataset, the DeepONet architecture, and the loss function during supervised learning. The data is split into a training set with $\approx 90$% of the input vectors (61 of the 68 $\varvec{c}$) and a validation set with the remaining $\approx 10$% (7 different $\varvec{c}$). Since the output at the sensor position is $\mathcal {M}_{\scriptscriptstyle {D}}(\varvec{c}) \in \mathbb {R}^{76\times 1}$, the training and validation sets comprise 4636 and 532 points, respectively. The training points are used for supervised learning, and the validation points are used to compute the generalization error on unseen data during training. The input layer of the DeepONet includes a mapping from the 52-dimensional $\varvec{c}$ to the 10-dimensional $\widetilde{\varvec{w}}$. This step enhances convergence during training, consistent with recent work by Floryan and Graham^55^ and Kontolati et al.^47^, where the dimension reduction in the data space was shown to facilitate learning. For example, Kontolati et al.^47^ deployed an autoencoder of the form $\varvec{w} = \phi ^{-1}(\varvec{c})$ as input to the DeepONet, introducing the so-called Latent DeepONet or L-DeepONet. Since we know the map $\varvec{c} = \phi (\varvec{w}_0 + \widetilde{\textbf{W}}\widetilde{\varvec{w}})$ by construction, we can explicitly compute $\widetilde{\varvec{w}}$ at the input layer of the DeepONet. Training required $\approx 2$ h of wall-clock time, on a desktop with NVIDIA RTX A4000 GPU, 32GB of RAM, and an Intel Xeon W-1250 CPU. On the same desktop, the evaluation of $\mathcal {M}_{\scriptscriptstyle {D}}$ requires $\mathcal {O}(0.01)$ seconds. Our DeepONet reaches a mean-square validation error, $\overline{\varepsilon }_{\scriptscriptstyle {D}}$, of $\approx 5\%$ (Fig. 7), which is also a measure of the average error of the DeepONet for the prediction of unseen in-distribution $\varvec{c}$.

### Searching the parameter space

Replacing $\mathcal {M}_{\scriptscriptstyle {D}}(\varvec{c})$ with $\mathcal {M}_{\scriptscriptstyle {S}}(\varvec{c})$ into Eq. (2) gives the cost function $\mathcal {J}_{\scriptscriptstyle {S}}(\varvec{w})$. DeepONet $\mathcal {M}_{\scriptscriptstyle {D}}$ is a meta-model for direct numerical simulations $\mathcal {M}_{\scriptscriptstyle {S}}$, and therefore $\mathcal {M}_{\scriptscriptstyle {D}}(\varvec{c}) = \mathcal {M}_{\scriptscriptstyle {S}}(\varvec{c}) + \varvec{\varepsilon }$ where $\varvec{\varepsilon }$ is the error. The associated cost functions for data assimilation are therefore related according to,

16 $$\begin{aligned} \mathcal {J}_{\scriptscriptstyle {D}}(\varvec{w})= \mathcal {J}_{\scriptscriptstyle {S}}(\varvec{w})- 2(\varvec{m}-\mathcal {M}_{\scriptscriptstyle {S}}(\varvec{c}))^{\top }\varvec{\Sigma }_{\varvec{m}}^{-1}\varvec{\varepsilon } + \varvec{\varepsilon }^{\top }\varvec{\Sigma }_{\varvec{m}}^{-1}\varvec{\varepsilon }. \end{aligned}$$

In effect, $\mathcal {J}_{\scriptscriptstyle {D}}(\varvec{w})$ is a noisy representation of $\mathcal {J}_{\scriptscriptstyle {S}}(\varvec{w})$. Since derivatives of $\mathcal {J}_{\scriptscriptstyle {D}}(\varvec{w})$ amplify erroneous oscillations in this approximation, use of gradient-based methods to minimize $\mathcal {J}_{\scriptscriptstyle {D}}(\varvec{w})$ is fraught with difficulty. A gradient-free approach much still be robust to noise in the DeepONet predictions, which can lead to an oscillatory cost landscape. For these reasons, we adopt an enhanced Nelder-Mead simplex algorithm^48^. The method is based on the evaluation of $\mathcal {J}_{\scriptscriptstyle {D}}(\varvec{w})$ on the vertices of a simplex, is gradient-free, and is robust to noise. Since the computational cost of the original Nelder-Mead algorithm scales with the dimension of the search space, the version deployed here includes adaptive parameters to maintain computational efficiency, also in a ten-dimensional search space. Therefore, it is possible to navigate the noisy $\mathcal {J}_{\scriptscriptstyle {D}}(\varvec{w})$ to converge to a solution of the minimization problem,

17 $$\begin{aligned} \min _{\varvec{w}} \mathcal {J}_{\scriptscriptstyle {D}}(\varvec{w}). \end{aligned}$$

The solutions to the data-assimilation problem are accurate up to the validation error of the DeepONet. On average, each solution requires $\mathcal {O}(3000)$ evaluations of $\mathcal {M}_{\scriptscriptstyle {D}}$, for a total time to solution of $\mathcal {O}(2)$ minutes. This average was computed by solving $392$ different data-assimilation problems which are discussed in the Results section, each with independent measurements $\varvec{m}$.

## Data Availability

Data sets generated during the current study are available from the corresponding author on reasonable request. Source codes for data generation and analysis are available at https://github.com/pmorra/FastDA.
